# Supplementary material for: Effect of sleep quality on vision-related quality of life in a sample of Qassim University students: an ocular surface health approach
Source: PeerJ. 2025 Aug 4;13:e19801. doi: 10.7717/peerj.19801 (PMC12330819; doi:10.7717/peerj.19801)
Supplement: Supplemental Information 2 [file peerj-13-19801-s002.doc]

STROBE Statement—Checklist of items that should be included in reports of ***cross-sectional studies***

|  | Item No | Recommendation |
| --- | --- | --- |
| **Title and abstract** | 1 | 1. Indicate the study’s design with a commonly used term in the title or the abstract   Cross- sectional study as stated in the Abstract line 21, and in Material and methods, Participants: line 94 |
|  |  | 1. Provide in the abstract an informative and balanced summary of what was done and what was found   Provided in the abstract on page 1 in methods and results sections |
| Introduction | | |
| Background/rationale | 2 | Explain the scientific background and rationale for the investigation being reported  Included in the introduction on page 2, rationale in line 61-63 |
| Objectives | 3 | State specific objectives, including any prespecified hypotheses  Included in the introduction lines 88-89 |
| Methods | | |
| Study design | 4 | Present key elements of study design early in the paper  Included in Page 3 |
| Setting | 5 | Describe the setting, locations, and relevant dates, including periods of recruitment, exposure, follow-up, and data collection  Included in Participants section of material and methods, lines 104-105. Follow-up is not applicable. |
| Participants | 6 | 1. Give the eligibility criteria, and the sources and methods of selection of participants   Provided in participants section, lines 103-104. |
| Variables | 7 | Clearly define all outcomes, exposures, predictors, potential confounders, and effect modifiers. Give diagnostic criteria, if applicable  Provided in each section of material and methods: Ocular examination, Ocular surface disease index, sleep quality assessment, and vision-related quality of life. Pages 3 and 4. |
| Data sources/ measurement | 8* | For each variable of interest, give sources of data and details of methods of assessment (measurement). Describe comparability of assessment methods if there is more than one group  References for each method or questionnaire used is provided |
| Bias | 9 | Describe any efforts to address potential sources of bias  Included in line 139. |
| Study size | 10 | Explain how the study size was arrived at  Included in Material and methods, lines 94-99. |
| Quantitative variables | 11 | Explain how quantitative variables were handled in the analyses. If applicable, describe which groupings were chosen and why  Included in data analysis section, lines 148-151. |
| Statistical methods | 12 | 1. Describe all statistical methods, including those used to control for confounding   Describes in Data analysis lines 148-151 |
|  |  | 1. Describe any methods used to examine subgroups and interactions   NA |
|  |  | 1. Explain how missing data were addressed   Explained in results, section: Quality of life related to vision and sleep quality metrics page 5 |
|  |  | 1. If applicable, describe analytical methods taking account of sampling strategy   NA |
|  |  | 1. Describe any sensitivity analyses   NA |
| Results | | |
| Participants | 13* | 1. Report numbers of individuals at each stage of study—eg numbers potentially eligible, examined for eligibility, confirmed eligible, included in the study, completing follow-up, and analysed   Included in study population section lines 160-163 |
|  |  | 1. Give reasons for non-participation at each stage   Included in study population section lines 160-163 |
|  |  | 1. Consider use of a flow diagram   NA |
| Descriptive data | 14* | 1. Give characteristics of study participants (eg demographic, clinical, social) and information on exposures and potential confounders   Included in study population section lines 160-163 |
|  |  | 1. Indicate number of participants with missing data for each variable of interest   Only had missing data in one part, mentioned in line 191. |
| Outcome data | 15* | Report numbers of outcome events or summary measures  Included in table 1, 2, and 3 |
| Main results | 16 | 1. Give unadjusted estimates and, if applicable, confounder-adjusted estimates and their precision (eg, 95% confidence interval). Make clear which confounders were adjusted for and why they were included   Mentioned in results page 5 and 6 |
|  |  | 1. Report category boundaries when continuous variables were categorized   NA |
|  |  | 1. If relevant, consider translating estimates of relative risk into absolute risk for a meaningful time period   NA |
| Other analyses | 17 | Report other analyses done—eg analyses of subgroups and interactions, and sensitivity analyses NA |
| Discussion | | |
| Key results | 18 | Summarise key results with reference to study objectives  Included on page 6 |
| Limitations | 19 | Discuss limitations of the study, taking into account sources of potential bias or imprecision. Discuss both direction and magnitude of any potential bias  Mentioned in conclusion section lines 247-250 |
| Interpretation | 20 | Give a cautious overall interpretation of results considering objectives, limitations, multiplicity of analyses, results from similar studies, and other relevant evidence  Discussed in pages 6 and 7. |
| Generalisability | 21 | Discuss the generalisability (external validity) of the study results  Discussed in pages 6 and 7. |
| Other information | | |
| Funding | 22 | Give the source of funding and the role of the funders for the present study and, if applicable, for the original study on which the present article is based  Provided in acknowledgment section page 7. |

*Give information separately for exposed and unexposed groups.

**Note:** An Explanation and Elaboration article discusses each checklist item and gives methodological background and published examples of transparent reporting. The STROBE checklist is best used in conjunction with this article (freely available on the Web sites of PLoS Medicine at http://www.plosmedicine.org/, Annals of Internal Medicine at http://www.annals.org/, and Epidemiology at http://www.epidem.com/). Information on the STROBE Initiative is available at www.strobe-statement.org.
